# Brain reward function in people who use cannabis: a systematic review

**DOI:** 10.3389/fnbeh.2023.1323609

**Published:** 2024-02-06

**Authors:** Emillie Beyer, Govinda Poudel, Stephanie Antonopoulos, Hannah Thomson, Valentina Lorenzetti

**Affiliations:** ^1^Neuroscience of Addiction and Mental Health Program, Healthy Brain and Mind Research Centre, School of Behavioural and Health Sciences, Australian Catholic University, Melbourne, VIC, Australia; ^2^Mary MacKillop Institute for Health Research, Australian Catholic University, Melbourne, VIC, Australia; ^3^Braincast Neurotechnologies, Melbourne, VIC, Australia; ^4^Turner Institute for Brain and Mental Health, School of Psychological Sciences, Monash University, Clayton, VIC, Australia

**Keywords:** cannabis, monetary incentive delay task (MIDT), reward processing, fMRI, neuroimaging, systematic review, functional neuroimaging

## Abstract

**Rationale:**

Cannabis is one of the most widely used psychoactive substances globally. Cannabis use can be associated with alterations of reward processing, including affective flattening, apathy, anhedonia, and lower sensitivity to natural rewards in conjunction with higher sensitivity to cannabis-related rewards. Such alterations have been posited to be driven by changes in underlying brain reward pathways, as per prominent neuroscientific theories of addiction. Functional neuroimaging (fMRI) studies have examined brain reward function in cannabis users via the monetary incentive delay (MID) fMRI task; however, this evidence is yet to be systematically synthesised.

**Objectives:**

We aimed to systematically integrate the evidence on brain reward function in cannabis users examined by the MID fMRI task; and in relation to metrics of cannabis exposure (e.g., dosage, frequency) and other behavioural variables.

**Method:**

We pre-registered the review in PROSPERO and reported it using PRISMA guidelines. Literature searches were conducted in PsycINFO, PubMed, Medline, CINAHL, and Scopus.

**Results:**

Nine studies were included, comprising 534 people with mean ages 16-to-28 years, of which 255 were people who use cannabis daily or almost daily, and 279 were controls. The fMRI literature to date led to largely non-significant group differences. A few studies reported group differences in the ventral striatum while participants anticipated rewards and losses; and in the caudate while participants received neutral outcomes. A few studies examined correlations between brain function and withdrawal, dosage, and age of onset; and reported inconsistent findings.

**Conclusions:**

There is emerging but inconsistent evidence of altered brain reward function in cannabis users examined with the MID fMRI task. Future fMRI studies are required to confirm if the brain reward system is altered in vulnerable cannabis users who experience a Cannabis Use Disorder, as postulated by prominent neuroscientific theories of addiction.

## 1 Introduction

Cannabis is one of the most widely used psychoactive substances globally, with over 219 million users (United Nations Office on Drugs and Crime, 2023). The regular use of cannabis can be associated with adverse psychosocial outcomes, including cannabis use disorders (CUD; Connor et al., 2021), mental health problems (Moore et al., 2007), impaired cognitive performance (Shrivastava et al., 2011), and hazardous behaviours (e.g., driving while intoxicated; Swift et al., 2010). Concerningly, cannabis use-related problems have been projected to rise with the increased accessibility and potency of cannabis products (Freeman et al., 2019). In order to develop effective preventative interventions in vulnerable people who use cannabis, it is important to understand the neurobiological mechanisms underlying cannabis use.

A key characteristic of regular cannabis use is altered processing of rewards (Pacheco-Colon et al., 2018). For example, people who use cannabis compared to controls show affective flattening, apathy, anhedonia, and decreased pleasure towards activities that are not related to cannabis use (Skumlien et al., 2021); as well as poorer cognitive performance during reward processing tasks (e.g., Iowa Gambling Task; Casey and Cservenka, 2020). In animal studies, repeated exposure to delta-9-tetrahydrocannabinol (THC; the main psychoactive compound of cannabis), leads to neuroadaptations within the brain's reward circuits, particularly within the mesolimbic dopamine system (Halbout et al., 2023).

Emerging functional neuroimaging evidence in human cannabis users has examined the neurobiology of reward processing (Balodis and Potenza, 2015; Skumlien et al., 2021). This work has been summarised by a recent high-quality systematic review (Skumlien et al., 2021). However, the review integrated evidence from varied functional Magnetic Resonance Imaging (fMRI) tasks (e.g., card guessing, coin toss, effort expenditure, and listening to music) with inconsistent cognitive load, complexity, and aspects of reward processing. As such, the findings could not be directly integrated. A separate review (Balodis and Potenza, 2015) synthesised findings from a specific reward processing fMRI task—the Monetary Incentive Delay (MID) task: the most consistently used and robust task to measure the function of the brain reward system (Oldham et al., 2018). However, the review included samples who use distinct substances (e.g., cannabis, alcohol, cocaine, nicotine; preventing the understanding of how the neurobiology of reward processing is affected by cannabis use specifically; Balodis and Potenza, 2015). Additionally, this review was published in 2015 and does not capture the most up-to-date literature (Balodis and Potenza, 2015). Furthermore, a systematic assessment of the *quality* of the fMRI literature of reward processing in cannabis users has yet to be conducted, which prevents a detailed interpretation of the evidence.

We aim to review the fMRI neuroimaging evidence to date that compared brain reward function between cannabis users and controls using the MID fMRI task. The secondary aim was to systematically synthesised the evidence on the association between brain reward function and metrics of cannabis use and related problems (e.g., dosage, frequency, and withdrawal), psychopathology symptom scores (e.g., anxiety, depression, and psychosis), and other variables (e.g., cognitive performance and other substance use).

## 2 Methods

This systematic review was pre-registered with PROSPERO (submitted 27/10/2022 and approved 17/11/2022; ID CRD42022354574) and was reported in accordance with the Preferred Reporting Items for Systematic Reviews and Meta-Analyses guidelines (PRISMA).

### 2.1 Literature search

A comprehensive electronic database search was conducted using PsycINFO, MEDLINE, CINAHL, Scopus, and PubMed on June 10, 2022. The search strategy is outlined in Supplementary material and employed three concepts related to: (i) cannabis; (ii) functional neuroimaging; and (iii) reward processing. Medical Subject Headings (MeSH) and keywords (synonyms) were combined with Boolean OR/AND operators for each concept and were searched across the title and abstracts of the returned articles. All full-text articles from the database searches were imported into the reference manager software Covidence (www.covidence.org), and duplicates were removed.

### 2.2 Inclusion and exclusion criteria

Studies were included if they: (i) examined human participants; (ii) were written in the English language; (iii) were full-text peer-reviewed articles; (iv) assessed a sample of people who consume cannabis, as defined by each study criterion; (v) included a non-cannabis user control group, as defined by each study criterion; (vi) measured brain function during the MID fMRI task, and (vii) compared brain function between cannabis and control groups.

Studies were excluded if they: (i) examined non-human participants; (ii) were published in languages other than English; (iii) were non-peer-reviewed (e.g., conference abstracts only); (iv) were non-empirical (e.g., single case reports, dissertations, editorials, corrigendum, book chapters, letters to the editor, reviews, and meta-analyses); (v) measured brain integrity using neuroimaging techniques other than fMRI, for example: structural magnetic resonance imaging (sMRI), computed tomography scan (CT), electroencephalogram (EEG), positron emission tomography (PET), single-photon emission computerised tomography (SPECT); (vi) studies that included tasks other than the MID fMRI task; (vii) measured brain function during acute cannabis intoxication; (viii) examined a sample of participants who endorsed significant substance use other than cannabis, alcohol, and nicotine at a group level; (ix) included a sample of participants who endorsed a diagnosis of axis I mental health disorders at a group level; or (x) examined a sample with diagnoses of neurological disorders or major medical conditions that affect the central nervous system [e.g., epilepsy, human immunodeficiency virus (HIV)].

### 2.3 Manuscript screening

The title, abstract, and then full text of all retrieved articles were screened by two researchers (E.B. and S.A.) in accordance with the above inclusion/exclusion criteria to determine eligibility. The final list of studies was compared and resolved by the researchers via consensus; if consensus could not be reached it was resolved by the senior author (V.L.). Additionally, the reference lists of all selected studies were cross-referenced to aid the inclusion of relevant work.

### 2.4 Data extraction

The following data were extracted:

(i) Study characteristics (e.g., first author, year of publication, and study location);
(ii) Participant characteristics (e.g., sample size, age, sex, and handedness);
(iii) Cannabis use level (e.g., dosage, frequency, age-onset, duration, and hours of abstinence);
(iv) CUD/dependence (e.g., instrument used, level, and presence/absence);
(v) Experiment characteristics (e.g., study design, dropouts, and reasons for dropouts);
(vi) fMRI reward task characteristics, such as instructions, cognitive function targeted by fMRI task, fMRI task parameters (e.g., duration), fMRI task design (e.g., counterbalanced order);
(vii) fMRI data analysis approach [e.g., whole brain, region of interest (ROI) based, seed-based, and relevant regions]; and measure of brain function (e.g., activity/connectivity).
(viii) The group differences in patterns of brain function (e.g., location, direction), and the relevant contrasts used (e.g., reward anticipation vs. neutral anticipation, reward anticipation vs. loss anticipation).
(ix) The association between the level of brain function (e.g., location/direction) during fMRI reward processing tasks in cannabis users and behavioural measures such as: cannabis use levels (e.g., dosage, frequency, age-onset, and duration), psychopathology symptom scores (e.g., anxiety, depression, and psychosis), and other measures (e.g., cognition and/or behavioural metrics).

### 2.5 Assessment of risk of bias of the reviewed literature

The Joanna Briggs Institute Critical Appraisal Checklist for Analytical Cross-Sectional Studies (Munn et al., 2014) was used to assess the quality of the included studies using eight distinct criteria. The criteria were: (i) Were the criteria for inclusion in the sample clearly defined?; (ii) Were the study subjects and the setting described in detail?; (iii) Was the exposure measured in a valid and reliable way—*this criteria was not applicable and therefore removed from the table*; (iv) Were objective, standard criteria used for measurement of the condition? (v) Were confounding factors identified? (vi) Were strategies to deal with confounding factors stated? (vii) Were the outcomes measured in a valid and reliable way? and (viii) Was appropriate statistical analysis used? Bias ratings were assessed for each included study based on the criteria above (E.B. and H.T.).

A score for each paper was generated, whereby each criterion was scored either 1 = endorsed, 0 = not endorsed, or 0.5 = partially endorsed. Subsequently, the quality of each paper was rated either high (i.e., a score of ≥8) moderate (i.e., a score between 4 and 7.5), or low (i.e., a score of ≤ 3.5).

### 2.6 PRISMA flowchart

**Figure 1:**
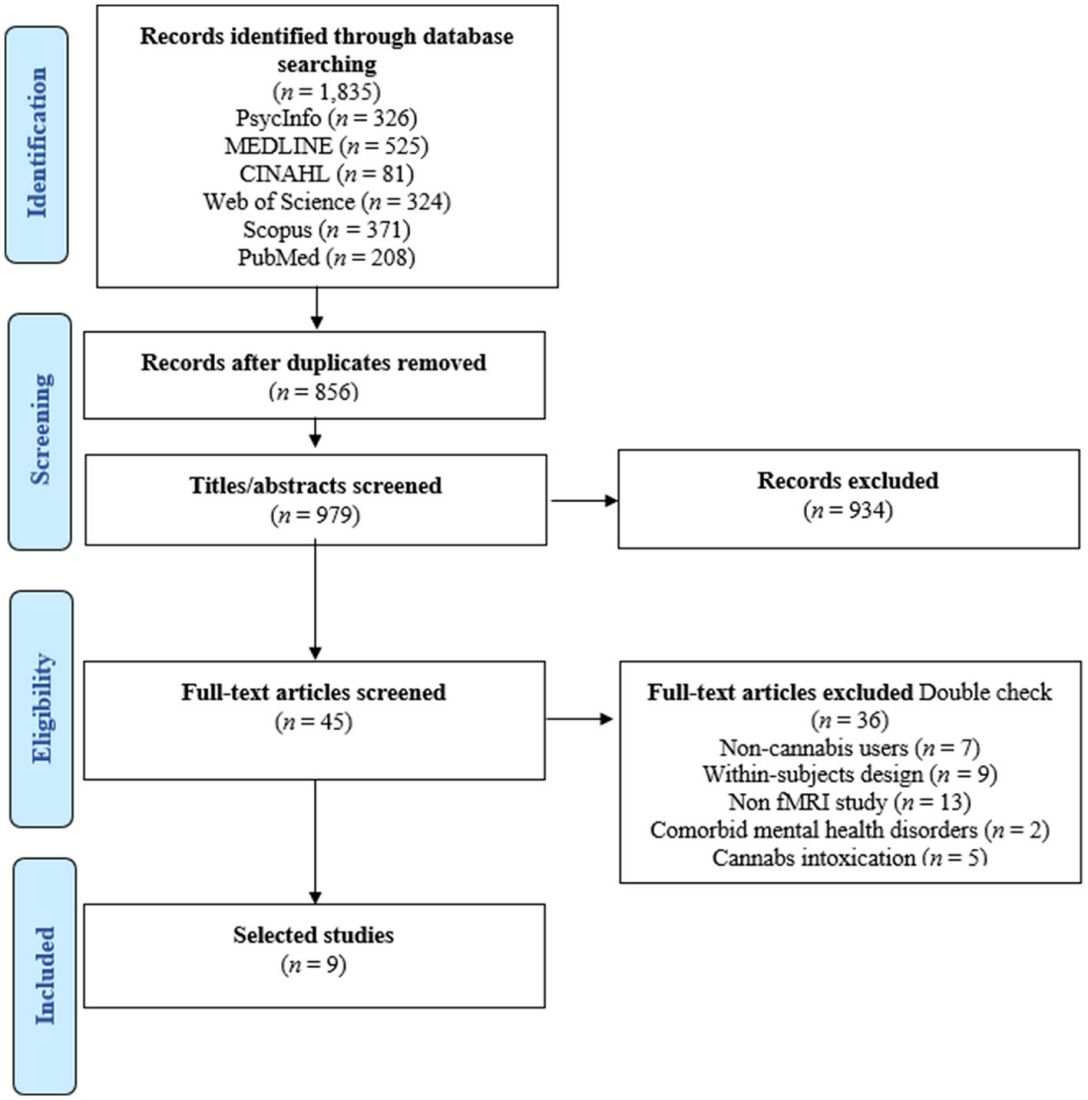
PRISMA flowchart.

Figure 1 illustrates the PRISMA flowchart. The initial database search produced 1,835 articles. After duplicates were removed, 979 titles and abstracts were screened based on the inclusion and exclusion criteria. Of these, 45 articles underwent full-text review, and 36 of them were not eligible for inclusion. Overall, nine studies were included in this systematic review (Nestor et al., 2010, 2020; van Hell et al., 2010; Filbey et al., 2013; Jager et al., 2013; Yip et al., 2014; Enzi et al., 2015; Karoly et al., 2015; Skumlien et al., 2022).

**Figure 2:**
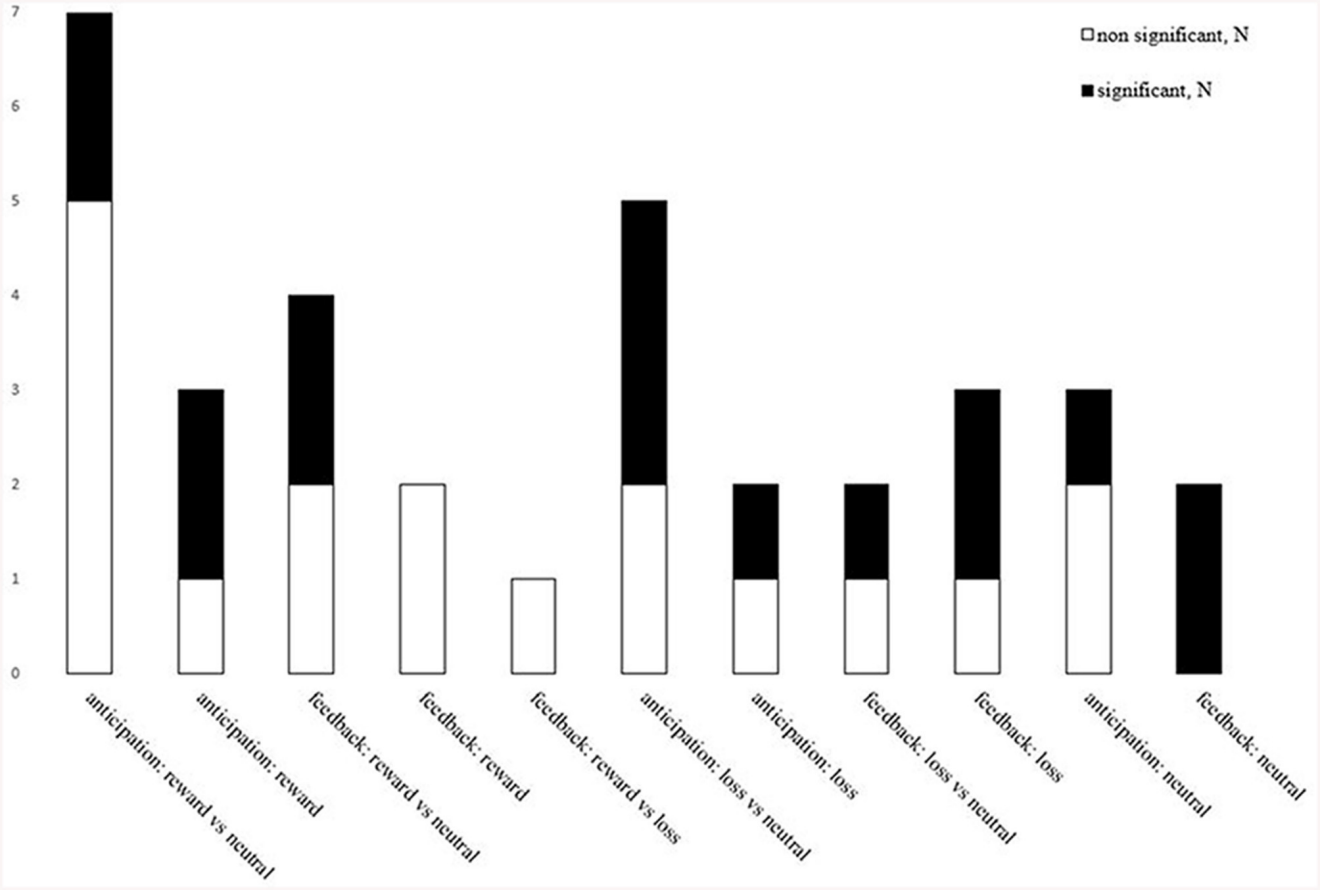
Overview of studies examining specific conditions/contrasts and of significant and non-significant findings. The total number of studies that found significant (black) and non-significant (white) findings between cannabis users compared to controls across the differing MID fMRI task contrasts. Anticipation: reward vs. neutral (seven studies; three significant): reward (three studies; two significant); loss vs. neutral (five studies; three significant); loss (two studies; one significant); neutral outcomes (three studies; one significant). Feedback: reward vs. neutral (four studies; two significant); reward (two studies; zero significant); reward vs. loss (one study; zero significant); loss vs. neutral (two studies; one significant); loss (three studies; two significant); neutral outcomes (two studies; two significant).

## 3 Results

### 3.1 Overview of studies and samples socio-demographic, substance use, and other characteristics

#### 3.1.1 Characteristics of the reviewed literature

Table 1 overviews the characteristics of the nine studies included in the review (Nestor et al., 2010, 2020; van Hell et al., 2010; Filbey et al., 2013; Jager et al., 2013; Yip et al., 2014; Enzi et al., 2015; Karoly et al., 2015; Skumlien et al., 2022). All studies were published in the past 13 years, between 2010 and 2022.

**Table 1:** Overview of demographics and cannabis use characteristics for the reviewed samples.

**References**	**Sub-groups**	**Sample** ***N*** **total (female) Age**, ***yrs***				**Cannabis use level**				**Behavioral group differences in MIDT performance**		
		**Cannabis**	**Control**	**Cannabis**	**Control**	**Age onset, yrs**	**Duration, yrs**	**Days/mo**	**Dosage**	**Abstinence duration**	**Accuracy**	**Reaction time**
Skumlien et al. (2022)	Adolescent	32 (16)	31 (15)	17 (1)	17 (0)	15 (1)	-	12 (8)	1 (1) *grammes p/w*	44 h	CB = HC (% correct) *No group-by-age interaction*	CB = HC *No group-by-age interaction*
	Adults	31 (14)	31 (16)	28 (1)	27 (1)	18 (3)	-	16 (8)		42 h	-	-
Nestor et al. (2020)		18 (1)	18 (1)	17 (0)	16 (0)	13 (0)	-	-	44 (9) *joints p/w*	Before scan	CB > HC CB = HC for relative motivational value (% accuracy during reward or loss/neutral)	CB = HC
Enzi et al. (2015)		15 (0)	15 (0)	26 (3)	27 (4)	16 (3)	8 (3)	-	13 (7) *joints p/w*	24 h	-	CB = HC
Karoly et al. (2015)	No-nicotine	14 (3)	38 (14)	16 (1)	16 (1)	13 (2)	-	20 (9)	98 (77) *hits p/w*	3 h	CB = HC & CB+tobacco, CB+alcohol +tobacco, tobacco, alcohol (% hits)	CB = HC and CB+tobacco, CB+alcohol +tobacco, tobacco, alcohol
	Nicotine	17 (4)	34 (13)	16 (1)	16 (1)	11 (2)	-	25 (7)	133 (175) *hits p/w*			
Yip et al. (2014)		20 (0)	20 (0)	27 (2)	29 (2)	13 (0)	14 (3)	16 (3)	-	24 h or 21 days	CB = HC (% correct hits)	CB = HC
Filbey et al. (2013)		59 (13)	27 (22)	23 (6)	30 (10)	15 (3)	9 (6)	28 (4)	-	72 h	CB = HC (% correct) CB = HC ($ won/lost)	CB = HC In CB, reward <loss, with $5 incentive
Jager et al. (2013)		21 (0)	24 (0)	17 (1)	17 (1)	13 (2)	-	-	15 (16) *joints p/w*	5 wks	CB = HC ($ won)	CB > HC, trend
Nestor et al. (2010)		14 (2)	14 (3)	22 (1)	23 (1)	16 (0)	6 (1)	20 (3)	16 (3) *joints p/w*	108 h	CB = HC (% correct)	CB = HC
van Hell et al. (2010)	Nicotine	14 (1)	14 (3)	24 (4)	25 (5)	-	-	-	11 (8) *joints p/w*	1 wk	CB = HC ($ won)	CB = HC
	Non-nicotine		13 (2)		24 (3)	-	-	-				

Hrs, hours; MIDT, monetary incentive delay task; m/o, months; p/w, per week; SD, standard deviation; wk, week; yrs, years.

Findings from Nestor et al. (2010), van Hell et al. (2010), Filbey et al. (2013), and Nestor et al. (2020) accounted for trial type (e.g., reward, loss).

#### 3.1.2 Overview of demographics of the reviewed samples

The reviewed samples comprised 534 participants (143 female and 391 males). Of these, 255 participants were people who use cannabis, and 279 were controls, with sample sizes ranging from 28 to 186. The average of the mean age of the samples was 22 years (range 16–28 years). The samples included both adolescents aged <18 years (three studies; Jager et al., 2013; Karoly et al., 2015; Nestor et al., 2020) and adults aged 18+ years (five studies; Nestor et al., 2010; van Hell et al., 2010; Filbey et al., 2013; Yip et al., 2014; Enzi et al., 2015) or both (1 study; Skumlien et al., 2022). Males were overrepresented in eight of the nine studies, and three studies recruited males only. See Supplementary material for recruitment sources and location of the reviewed studies.

#### 3.1.3 Overview of levels of cannabis use

Table 1 outlines the level of cannabis consumption in the examined samples. The mean *age of cannabis use onset* was 16 years (range; 13-to-18 years). The level of mean *cannabis dosage* varied across the reviewed samples: 20 joints/week (range; 13–44 (e.g., joints; joints/week), 1 gramme/week (Skumlien et al., 2022); and 14 cannabis hits/day (Karoly et al., 2015). All samples included a group of people with current cannabis use, except for two studies, where cannabis groups were abstinent for ~21 days (Yip et al., 2014) or 5 weeks (Jager et al., 2013). Two studies included additional control participants who used nicotine and participants who did not use nicotine (van Hell et al., 2010; Karoly et al., 2015).

### 3.2 Overview of methodologies used in the reviewed literature

#### 3.2.1 Characteristics of the MID fMRI task

All nine studies used a modified version of the original MID fMRI task, developed by Knutson et al. (2001). The basic structure of the task included the following stages in this order: (i) *a reward cue*, singling a potential “win,” “loss,” or “no outcome” cue, (ii) a *target stimulus* where participants press a button to try to win or to avoid losing money, and (iii) a *feedback* stage where participants received feedback on either winning money, avoiding losing money, or losing money.

To note, studies used the terms win/reward or feedback/receipt interchangeably. As such, we use the terms “anticipation of reward/loss/neutral” and “feedback of reward/loss/neutral” for consistency and readability. Additional information on the parameters of the MID fMRI task is summarised in Supplementary material.

#### 3.2.2 Overview of fMRI data analysis methods

The studies used different fMRI data analysis methods. They included: exploratory whole-brain analysis (three studies; Nestor et al., 2010; Filbey et al., 2013; Enzi et al., 2015), a priori region of interest (ROI) analysis focused on hypothesis-driven areas (two studies; Yip et al., 2014; Karoly et al., 2015). A total of two studies focused on the striatal ROIs: the ventral striatum (Yip et al., 2014; Skumlien et al., 2022). Other studies focused on the nucleus accumbens (NAcc; van Hell et al., 2010; Karoly et al., 2015), and caudate (Jager et al., 2013; Yip et al., 2014). Individual studies used other ROIs: the putamen (Jager et al., 2013), and the ventromedial prefrontal cortex (vmPFC; Skumlien et al., 2022). A total of three studies used both whole-brain and ROI approaches (van Hell et al., 2010; Yip et al., 2014; Skumlien et al., 2022). An individual study used graph theory ROI-to-ROI functional connectivity (Nestor et al., 2020).

### 3.3 Group differences in behavioural task performance

There were largely non-significant group differences in behavioural task performance, including accuracy and reaction times (Table 1). Only one of the nine studies reported that cannabis users were significantly more accurate than controls during loss vs. neutral trials (Nestor et al., 2020). One study found that cannabis users had a trend of slower reaction times than controls, during reward vs. neutral trials (Jager et al., 2013).

### 3.4 Brain functional differences during the MID fMRI task

**Figure 3:**
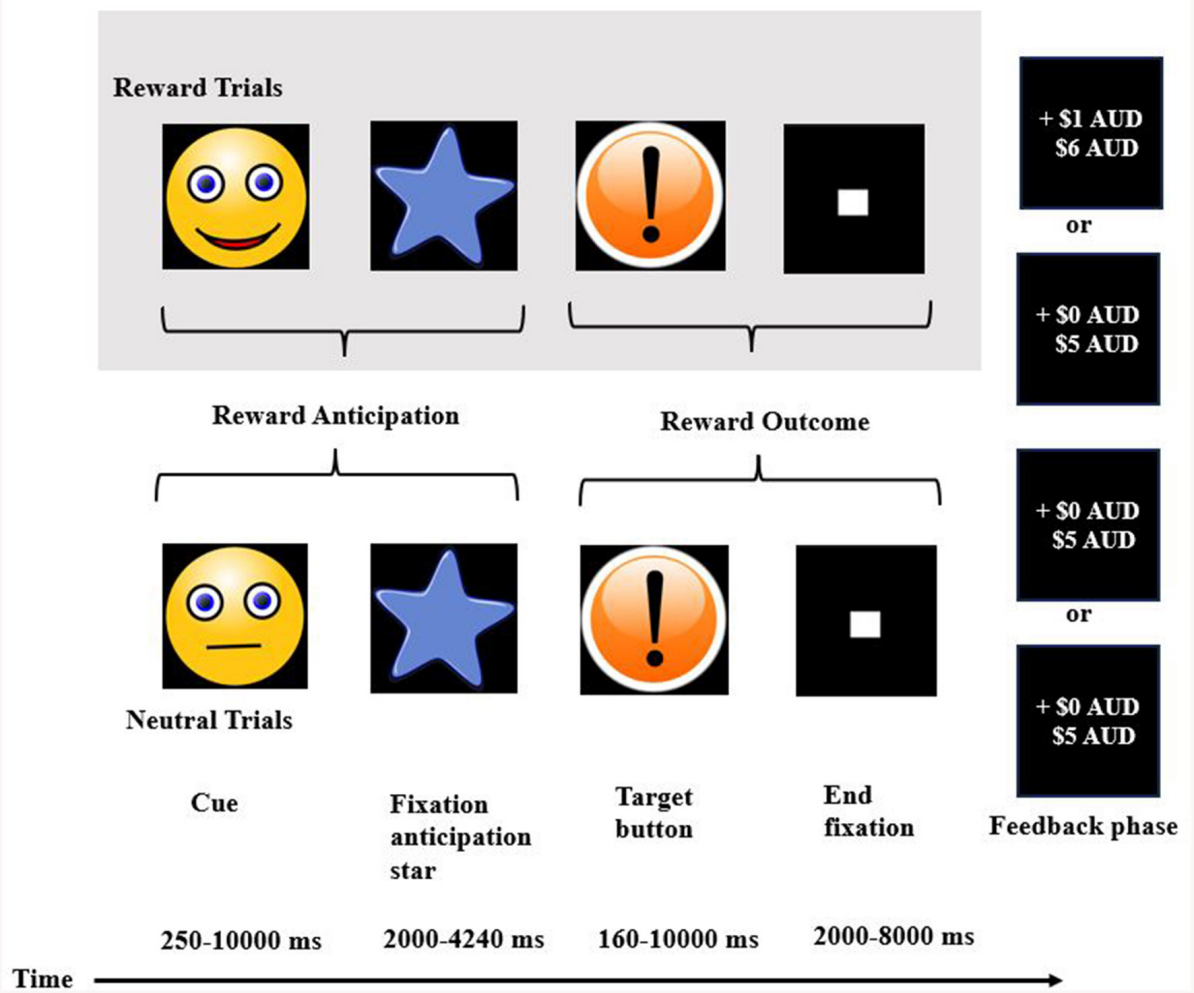
Example of the structure of the monetary incentive delay fMRI task.

This section overviews group differences in brain function during reward, loss, and neutral conditions (Figure 3). Out of 34 contrasts reported, 16 found significant group differences.

### 3.5 Group differences in brain function during reward trials

This section overviews group differences during the anticipation and receipt of rewards (Tables 2–4, respectively).

**Table 2:** Group differences in brain function during anticipation of *monetary rewards*.

**References**	**fMRI data analysis**		**Brain functional results**	
	**Type**	**Thresholding**	**Group examined/compared**	**Brain behaviour associations**
**Anticipation of** ***rewarding outcomes vs. neutral outcomes***				
Skumlien et al. (2022)	Whole brain	*p* < 0.001, *z* = 3.1, clusterwise corrected	CB = HC: both adults and adolescents	-
	ROI (ventral striatum, vmPFC)	-	CB = HC: both adults and adolescents *accounting for RT, depression (BDI), maternal education, use of alcohol, cigarette, and other drugs*	***Neg. corr***. CUD & ventral striatum ***Non-sig. corr***. CUDIT, frequency (day/wk), dosage (daily grammes), age onset (first use, weekly use), hours last use
Karoly et al. (2015)	ROI (NAcc)	-	CB > HC: NAcc CB+alcohol+tobacco > tobacco: NAcc CB = HC and alcohol, CB+tobacco, CB+tobacco+alcohol	-
Enzi et al. (2015)	Whole brain	*p* < 0.0015, *k* = 10, uncorrected	CB = HC	-
Yip et al. (2014)	ROI (ventral striatum)	-	CB = HC	***-***
Jager et al. (2013)	Whole brain	*p* < 0.05, FWE	CB = HC	-
Filbey et al. (2013)	Whole brain	*p* < 0.05, *z* = 1.96, clusterwise corrected	CB = HC	***Neg. corr***. withdrawal and OFC, ACC*, controlling for age onset and duration. **Sig. corr***. craving and lingual gyrus, MFG ***Non-sig. corr***. SCID
van Hell et al. (2010)	Whole brain	*p* < 0.05, *t* > 4.5, corrected for multiple comparisons	CB <HC: NAcc, caudate, putamen, inferior medial/superior frontal and cingulate CB > HC: middle temporal, para-hippocampus, cuneus CB <tobacco: middle temporal CB > tobacco: NAcc, medial frontal, cingulate	-
	ROI (NAcc)	-	CB+tobacco >HC: NAcc *accounting for N cigarettes*.	***Non-sig. corr***. N cigarettes and NAcc
**Anticipation of** ***rewarding outcomes***				
Nestor et al. (2020)	Whole brain	*z* > 2.3, FWE, *p* < 0.05	CB=HC	**-**
	ROI-to-ROI connectivity	*t* > 3.1, *p* < 0.05, FWE clusterwise corrected, permutation testing (5,000 permutations)	CB > HC: graph subnetwork of 63 edges between 46 nodes: NAcc, insula, PFC areas (lateral/medial PFC, OFC, frontal pole), temporal areas (amygdala, hippocampus/para-hippocampus, temporal pole/cortex, temporal); other regions (central opercular, posterior cingulate, parietal opercular, intra/supra-calcarine, supplementary motor, superior parietal, posterior division, fusiform)	**-**
	Graph theory	Distinct *k* thresholds (0.1 <*k* < 0.5, increments of 0.1), *bonferroni-corrected at p <0.004*	CB > HC: clustering coefficient CB <HC: path length, global efficiency	***Pos. corr***. age onset and clustering coefficient/global efficiency ***Neg. corr***. age onset and path length ***Non-sign. cor***. dosage (lifetime joints)
Jager et al. (2013)	ROI (caudate, putamen)	-	CB = HC	**-**
Nestor et al. (2010)	Whole brain	*p* < 0.05, clusterwise corrected	CB > HC: ventral striatum caudate, putamen; ventral putamen and lentiform nucleus: MFG, fusiform: cerebellum (declive of vermis) CB <HC: fusiform	***Pos. corr***. duration, dosage (lifetime joints) and ventral putamen, cingulate, cerebellum (declive of vermis); duration, dosage (lifetime joints) and MFG; cuneus, ventral striatum, putamen, cerebellum (declive of vermis) ***Neg. corr***. withdrawal and fusiform gyrus; duration, dosage (lifetime joints)/withdrawal and MFG ***Non-sig. corr***. craving, alcohol, other drug exposure

CB, cannabis users; HC, controls; N, number; Neg. corr, negative correlation; Non-sig. corr, non-significant correlation; Pos. corr, positive correlation; RT, randomised trial; wk, week. ACC, anterior cingulate cortex; BDI, Beck Depression Inventory; CUD, cannabis use disorder; CUIDT, cannabis use disorder identification test; MFG; middle frontal gyrus; NAcc, nucleus accumbens; OFC, orbitofrontal cortex; PFC, prefrontal cortex; SCID, structured clinical interview for DSM disorders.

**Table 3:** Group differences in brain function during the receipt of *monetary rewards*.

**References**	**fMRI data analysis**		**Brain functional results**	
	**Type**	**Thresholding**	**Group examined/compared**	**Brain behaviour associations**
**Receipt of** ***rewarding outcomes vs. neutral outcomes***				
Skumlien et al. (2022)	Whole brain	*p* < 0.001, *z* = 3.1, cluster-wise corrected	CB > HC: frontal areas, parietal (supramarginal, angular) *in both adults and adolescents and age group*	-
	ROI (ventral striatum, vmPFC)	-	CB = HC: both adults and adolescents *accounting for RT, depression (BDI), maternal education, use of alcohol, cigarette, other drugs*	***Non-sig. corr***. CUD, CUDIT, frequency (day/wk), dosage (daily grammes), hours last use, age onset (first use, weekly use)
Enzi et al. (2015)	Whole brain	*p* < 0.001, *k* = 10	CB = HC	-
Yip et al. (2014)	ROI (ventral striatum)	-	CB = HC: ventral striatum *across all win outcomes considered together ($5 vs. $0 and $1 vs. $0), & separately*.	-
van Hell et al. (2010)	Whole brain	*p* < 0.05, *t* > 4.5, corrected for multiple comparisons	CB > HC: ventral striatum (caudate, putamen) frontal areas (inferior/medial frontal, cingulate, and precentral), temporal areas (middle temporal, parahippocampus), parietal (postcentral, precuneus), middle occipital. CB <HC: frontal areas (middle frontal, and claustrum), cingulate/posterior cingulate, temporal gyrus (middle/superior), parietal (postcentral, inferior parietal lobule), occipital (lingual, fusiform, inferior occipital).	-
	ROI (NAcc)	-	CB = tobacco = HC: NAcc	***Non-sig. corr***. cigarettes and NAcc
**Receipt of** ***rewarding outcomes***				
Enzi et al. (2015)	Whole brain	*p* < 0.001, *k* = 10, uncorrected	CB = HC	-
Filbey et al. (2013)	Whole brain	*p* < 0.05, *z* = 2.3	CB = HC	-

CB, cannabis users; HC, controls; N, number; Non-sig. corr, non-significant correlation; RT, randomised trial; wk, week. BDI, Beck Depression Inventory; CUD, cannabis use disorder; CUIDT, cannabis use disorder identification test; NAcc, nucleus accumbens; SCID, structured clinical interview for DSM disorders.

**Table 4:** Group differences during the *anticipation of monetary losses*.

**References**	**fMRI data analysis**		**Brain functional results**	
	**Type**	**Thresholding**	**Group examined/compared**	**Brain behaviour associations**
**Anticipation of** ***loss outcomes vs. neutral outcomes***				
Enzi et al. (2015)	Whole brain	*p* < 0.0015, *k* = 10, uncorrected	CB = HC	-
Karoly et al. (2015)	ROI (NAcc)	-	CB = HC and CB+tobacco, CB+alcohol, CB+tobacco+alcohol	-
Yip et al. (2014)	ROI (ventral striatum)	-	CB > HC: ventral striatum	**-**
Filbey et al. (2013)	Whole brain	*p* < 0.05, *z* = 1.96, clusterwise corrected	CB = HC	***Neg. corr***. withdrawal and OFC, ventral striatum, amygdala and hippocampus, *controlling for age onset and duration. **Non-sig. corr***. SCID
Nestor et al. (2010)	Whole brain	*p* < 0.05, clusterwise corrected	CB > HC: ventral putamen	-
**Anticipation of** ***loss outcomes***				
Nestor et al. (2020)	Whole brain	*z* > 2.3, FWE, *p* < 0.05	CB = HC	-
	ROI-to-ROI connectivity	*t* > 3.1, *p* < 0.05, FWE cluster corrected, permutation testing (5,000 permutations)	CB = HC	-
	Graph theory	Distinct K thresholds (0.1 <*k* < 0.5, increments of 0.1), bonferroni-corrected at *p* < 0.004	CB = HC	***Non-sign. corr***. dose (lifetime joints), age onset
Nestor et al. (2010)	Whole brain	*p* < 0.05, clusterwise corrected	CB > HC: ventral putamen; cerebellum (declive of vermis)	***Non-sig. corr***. craving, withdrawal, alcohol and other drug exposure

CB, cannabis users; HC, controls; Neg. corr, negative correlation; Non-sig. corr, non-significant correlation; OFC, orbitofrontal cortex; SCID, structured clinical interview for DSM disorders.

#### 3.5.1 Group differences while anticipating monetary rewards vs. neutral outcomes

Seven studies compared groups by brain function during anticipation of *monetary rewards vs. neutral outcomes* (van Hell et al., 2010; Filbey et al., 2013; Jager et al., 2013; Yip et al., 2014; Enzi et al., 2015; Karoly et al., 2015; Skumlien et al., 2022). Of these, five studies reported non-significant group differences. Only two studies reported significantly different brain function in the NAcc (van Hell et al., 2010; Karoly et al., 2015). An individual study reported group differences in other prefrontal-striatal regions (e.g., superior frontal cortex; van Hell et al., 2010).

Only three studies examined the association between brain function during *reward anticipation vs. neutral anticipation*, and cannabis use metrics. Two of the three studies reported significant correlations in opposite directions (Filbey et al., 2013; Skumlien et al., 2022). Two studies reported negative correlations between CUD symptoms, withdrawal, and brain function across the prefrontal-striatal regions, and one significant positive correlation between craving and middle frontal/lingual function (Table 2).

#### 3.5.2 Group differences while anticipating monetary rewards

Two of three studies that examined group differences while participants *anticipated monetary rewards*, reported significant results in the ventral striatum (Nestor et al., 2010, 2020). One study reported different brain function in additional regions (e.g., insula, PFC, amygdala; Nestor et al., 2020).

Two studies reported significant associations between brain function during *reward anticipation* and the age of cannabis use onset (e.g., cluster efficiency), dosage (e.g., ventral striatum), and withdrawal (e.g., medial frontal gyrus; Nestor et al., 2010, 2020) (Table 2).

#### 3.5.3 Group differences while receiving monetary rewards vs. neutral feedback

Four studies examined group differences while participants *received rewards vs. neutral outcomes* (van Hell et al., 2010; Yip et al., 2014; Enzi et al., 2015; Skumlien et al., 2022). Of these, two studies reported that cannabis users had altered brain function in prefrontal, striatal, and parietal regions (e.g., caudate, putamen, MFG, and precuneus); and no study reported significant correlations between CUD symptoms, cannabis craving, and nicotine use (Table 3).

#### 3.5.4 Group differences while receiving monetary rewards

No study reported group differences in brain function while participants received *monetary rewards*; or explored correlations with behavioural measures (Table 3).

### 3.6 Group differences in brain function during monetary losses

This section overviews group differences in brain function during the anticipation and receipt of losses (Table 4).

#### 3.6.1 Group differences while anticipating monetary losses vs. neutral outcomes

Only two of five studies found group differences during the *anticipation of monetary losses vs. neutral outcomes* (Nestor et al., 2010; Filbey et al., 2013; Yip et al., 2014; Enzi et al., 2015; Karoly et al., 2015), including greater activity in the ventral striatum (Yip et al., 2014), and the ventral putamen (Nestor et al., 2010).

A single study found that greater withdrawal correlated with lower activation in OFC, ventral striatal, and temporal regions (Filbey et al., 2013) (Table 4).

#### 3.6.2 Group differences while anticipating monetary losses

Only one of two studies that examined brain function during *anticipation of monetary losses* (Nestor et al., 2010, 2020), reported greater brain activity in distinct regions (e.g., putamen, cerebellum; Nestor et al., 2010). Neither study found significant correlations between cannabis use metrics and brain function (Table 4).

#### 3.6.3 Group differences while receiving monetary losses vs. neutral feedback

One of two studies reported different brain function during *monetary loss feedback vs. neutral feedback* (Yip et al., 2014; Enzi et al., 2015), including greater activity of the ventral striatum, thalamus, and brainstem (Yip et al., 2014). Neither study examined brain-behaviour correlations (Table 5).

**Table 5:** Brain function group difference between cannabis users and controls while people received *monetary losses*.

**References**	**fMRI data analysis**		**Brain functional results**	
	**Type**	**Thresholding**	**Group examined/compared**	**Brain behaviour associations**
**Receipt of losses vs. receipt of neutral outcomes**				
Enzi et al. (2015)	Whole brain	*p* < 0.001, *k* = 10	CB = HC	-
Yip et al. (2014)	Whole brain	*p* < 0.05, FWE	CB > HC: ventral striatum; caudate, putamen, thalamus, and brainstem	-
	ROI (ventral striatum)	-	CB > HC: ventral striatum	-
	ROI (caudate)	-	CB > HC: caudate CB = abstinent CB and HC: caudate	-
**Receipt of losses**				
Enzi et al. (2015)	Whole brain	*p* < 0.001, *k* = 10, uncorrected	CB > HC: caudate, inferior frontal gyrus	***Pos. corr***. dose (lifetime joints) and caudate ***Non-sig. corr***. THC-COOH, age onset, dosage (lifetime joints) and abstinence.
Filbey et al. (2013)	Whole brain	*p* < 0.05, *z* = 2.3	CB = HC	-
Nestor et al. (2010)	Whole brain	*p* < 0.05, clusterwise corrected	*Loss of 50c:* CB > HC: superior parietal lobule CB <HC: insula, precentral gyrus	***Neg. corr***. dose (lifetime joints) and superior parietal lobule
	Whole brain	*p* < 0.05, clusterwise corrected	*Feedback that a loss of 5c was avoided:* CB <HC: insula	***Non-sig. corr***. craving, withdrawal, alcohol, and other drug exposure.

CB, cannabis users; HC, controls; Neg. corr, negative correlation; Non-sig. corr, non-significant correlation; Pos. corr, positive correlation; THC-COOH, carboxy-Δ9-tetrahydrocannabinol.

#### 3.6.4 Group differences while receiving monetary losses

Two of three studies found that cannabis users had greater activity when receiving *monetary losses*, in distinct regions (e.g., caudate, inferior frontal gyrus and superior parietal lobule; Filbey et al., 2013; Enzi et al., 2015). Similarly, two of three studies found that brain function correlated with cannabis dosage (i.e., caudate, and superior parietal lobule; Nestor et al., 2010; Enzi et al., 2015).

One study reported non-significant group differences in brain function during *reward feedback* compared to *loss feedback* (Enzi et al., 2015) (Table 5).

### 3.7 Group differences in brain function during neutral trials

This section overviews group differences during anticipation and receipt of neutral outcomes (Table 6).

**Table 6:** Brain function group difference between cannabis users and controls during neutral trials.

**References**	**fMRI data analysis**		**Brain functional results**	
	**Type**	**Thresholding**	**Group examined/compared**	**Brain behaviour associations**
**Anticipation of neutral outcomes**				
Nestor et al. (2020)	Whole brain	*z* > 2.3, FWE, *p* < 0.05	CB = HC	-
	ROI-to-ROI connectivity	*t* > 3.1, *p* < 0.05, FWE clusterwise corrected, permutation testing (5,000 permutations)	CB = HC	-
	Graph theory	Distinct *k* thresholds (0.1 <*k* < 0.5, increments of 0.11 *Bonferronioni –corrected*	CB = HC	***Non-sign. corr***. onset age and dose (lifetime joints)
Jager et al. (2013)	ROI (caudate, putamen)	-	CB > HC: caudate (trend) and putamen (trend)	***Neg. corr***. onset age and caudate ***Non-sig. corr***. onset age and putamen; dose (lifetime/past year joints) and caudate/putamen
Nestor et al. (2010)	Whole brain	*p* < 0.05, clusterwise corrected	CB = HC	-
**Receipt of neutral outcomes**				
Enzi et al. (2015)	Whole brain	*p* < 0.001, *k* = 10, uncorrected	CB > HC: caudate, IFG	***Non-sig. corr***. onset age, dose (lifetime joints), THC-COOH, and abstinence
Nestor et al. (2010)	Whole brain	*p* < 0.05, clusterwise corrected	*Loss of 50c:* CB > HC: caudate, IFG, cingulate, MFG, SFG, STG, parahippocampus, precentral, postcentral, cuneus, culmen, middle occipital, and brainstem	-
	Whole brain	*p* < 0.05, clusterwise corrected	CB > HC: caudate, IFG, cingulate, parahippocampus, uncus, and cerebellum. CB <HC: paracentral lobule *Neutral win (win 50c)*	

CB, cannabis users; HC, controls; Neg. corr, negative correlation; Non-sig. corr, non-significant correlation; IFG, inferior frontal gyrus; MFG, middle frontal gyrus; THC-COOH, carboxy-Δ^9^-tetrahydrocannabinol.

#### 3.7.1 Group differences while anticipating neutral outcomes

Two of three studies found no significant group differences while anticipating neutral outcomes (Nestor et al., 2010, 2020). The other study reported trend-level greater activity of the putamen and caudate, and caudate activity correlated with earlier age of cannabis use onset (Jager et al., 2013). Other correlations led to non-significant results.

#### 3.7.2 Group differences while receiving neutral outcomes

Both studies that examined brain function while receiving *neutral outcomes* reported significant differences in the caudate and inferior frontal gyrus (IFG; Nestor et al., 2010; Enzi et al., 2015). The only study to examine brain-behaviour correlations reported non-significant results (Enzi et al., 2015).

### 3.8 Overview of brain regions most consistently reported to differ between groups

Overall, the most consistently reported finding was altered NAcc activation in two of seven studies during reward anticipation vs. neutral anticipation (van Hell et al., 2010; Karoly et al., 2015), followed by the ventral striatum during loss anticipation vs. neutral anticipation (two of three studies; Nestor et al., 2010; Yip et al., 2014), and the caudate while participants received neutral outcomes (two of two studies; Nestor et al., 2010; Enzi et al., 2015).

## 4 Discussion

The fMRI evidence that brain reward function is altered in people who use cannabis is limited using the MID task, and led to largely non-significant or mixed findings, in contrast with prominent neuroscientific theories of addiction (Robinson and Berridge, 2001; Koob and Volkow, 2016). Yet within this, the few studies that reported significant group differences consistently identified changes in striatal regions underlying reward processing: the ventral striatum during anticipation of *monetary rewards* and losses; and the caudate while receiving neutral outcomes. There was emerging and inconsistent evidence that reward striatal function correlated with cannabis exposure and related problems (e.g., withdrawal, dosage, and age of onset).

There was no evidence of altered brain function in cannabis users during reward anticipation (van Hell et al., 2010; Karoly et al., 2015) and receipt (Nestor et al., 2010; Enzi et al., 2015). These findings contrast neuroimaging evidence of consistent striatal alterations during anticipation of rewards, in other substances, and in behavioural addictions (e.g., to gambling, cocaine, alcohol; Balodis and Potenza, 2015).

The discrepancy between the reviewed body of work in cannabis users and findings on other substances may additionally be attributed to distinct methodological issues. First, exposure to cannabis vs. other substances may exert a different effect on mesocorticolimbic pathways due to their distinct psychopharmacology (Oleson and Cheer, 2012). For example, exposure to cocaine robustly targets dopaminergic pathways to increase dopamine (Juarez and Han, 2016). Instead, THC induces only a modest dopamine increase (of 3.65%) within the limbic striatum; which is below the threshold of 5% of test-retest variability, meaning that the increase reported might reflect measurement error (Bossong et al., 2015). Thus, regular cannabis use might affect the reward circuitry less so than other drugs known to affect dopaminergic fronto-striatal pathways (e.g., cocaine). Alternatively, unmeasured variables entrenched with cannabis use may explain the emerging alterations in a portion of the studies. The variables might include: greater cannabis use related problems (Lorenzetti et al., 2016, 2020; Chye et al., 2019; Rossetti et al., 2021), poly-substance use (e.g., nicotine, alcohol; Brody et al., 2004), substance-related psychosocial variables (Jackson et al., 2020) and young age (mean = 22 years, range = 16–28 years in the reviewed literature), which may protect from the adverse impact of cannabis use on the brain (Lorenzetti et al., 2023). Notably, a paucity of studies measured potential moderators in relation to brain function (e.g., sex; Becker and Chartoff, 2019), and more evidence is required to test these notions.

Preliminary findings suggest that prefrontal (e.g., OFC, MFG) brain function during *reward anticipation* is associated with cannabis withdrawal (Nestor et al., 2010; Filbey et al., 2013). However, these correlations emerged in studies that reported no group difference in brain reward function. If greater withdrawal drove altered mesocorticolimbic function in cannabis users, it is possible that withdrawal levels were not severe enough in the reviewed samples to drive observable group differences. Indeed, most studies examined current cannabis users who were abstinent from cannabis on average between 1 and 7 days prior to testing. Further, withdrawal was largely unmeasured in the reviewed samples and is a key characteristic of cannabis dependence/CUD. Thus, future work should systematically examine the role of cannabis dependence/CUD and withdrawal in the neurobiology of reward processing.

Despite early findings of group differences in brain reward function, overall there was a lack of differences in behavioural task performance (e.g., reaction times, accuracy). Thus, emerging brain changes might have been insufficient to cause behavioural alterations, or might reflect a compensatory mechanism whereby cannabis users had to engage greater neural resources to perform similarly to controls (Mikulskaya and Martin, 2018).

There was emerging evidence that loss anticipation/receipt was underscored by different prefrontal-striatal function (e.g., IFG, putamen/caudate); and in correlation with greater dosage and earlier cannabis use onset. There is a paucity of evidence examining these variables. Therefore, we are not yet able to draw conclusions on the neurobiology of loss processing in cannabis users (e.g., anticipating, receiving, and avoiding negative outcomes); and in comparison with findings from normative samples (Oldham et al., 2018), and from samples who use substances other than cannabis (Morie et al., 2016).

Overall, the findings herein do not align with robust alterations of mesocorticolimbic pathways while processing non-drug related rewards and losses, as shown in substances other than cannabis and as postulated by prominent neuroscientific theories of addiction (e.g., incentive salience theory; Robinson and Berridge, 2001).

### 4.1 Limitations of the literature

The findings from the reviewed literature need to be interpreted in light of methodological limitations. First, the reviewed evidence is cross-sectional, and longitudinal work is required to determine if altered incentive salience predates or follows cannabis use onset. Second, to date, only nine fMRI studies used the MID task to examine brain reward function in people who use cannabis. The low number of studies precluded the running of a meta-analysis, which requires at least 17–20 studies employing an unbiased whole-brain approach (Muller et al., 2018). Instead, in the literature reviewed herein, the most consistently examined contrasts were reported only by five studies (i.e., *reward anticipation* vs. *neutral anticipation*) and three studies (e.g., *reward feedback vs. neutral feedback*; *loss anticipation vs. neutral anticipation*; and *loss feedback*) or <2 studies. Therefore, replication work is required to confirm how reward/loss processing plays a role in the neurobiology of cannabis use; and to explore which variables moderate such associations (e.g., presence of CUD/dependence, greater withdrawal, and exposure to cannabis and nicotine). Additionally, given the low number of studies, we could not systematically quantify differences in brain reward function between adolescents and adults. Furthermore, within the existing literature, there was no emerging evidence of consistent patterns of brain reward function between age groups. Moreover, one study which did directly compare adolescents vs. adults, found no differences in brain reward function (Skumlien et al., 2022). As such, future research is required to determine if age moderates group differences in brain reward function.

Third, assessment of CUD/cannabis dependence was lacking in half of the studies. Future work is required to demonstrate if processing non-drug related rewards affect the mesocorticolimbic circuitry differently in CUD/dependence vs. non-dependent use, as shown in other measures of neural integrity in cannabis users (Chye et al., 2019; Lorenzetti et al., 2020; Rossetti et al., 2021), and as postulated by neuroscientific theories of addiction (Robinson and Berridge, 2001; Koob and Volkow, 2016). Finally, assessment of metrics of exposure to cannabis/other substances and in relation to brain function is lacking. This issue precludes the understanding of which mechanisms may drive changes in reward brain function in people who use cannabis. To address this gap, future work should use robust metrics of substance use e.g., THC Unit, iCannToolkit (Freeman and Lorenzetti, 2020; Lorenzetti et al., 2022) and related problems using tools with diagnostic cutoffs (e.g., CUDIT; Myers et al., 2023).

### 4.2 Limitations of this review

The review focused on a single measure of reward processing i.e., the MID fMRI task. Perhaps, integrating other reward processing tasks, could have included other aspects of reward processing relevant to the neurobiology of cannabis use (e.g., reversal of learning contingencies with a reward learning task). Yet, the MID was the most consistently used fMRI reward processing task to date in cannabis users (and in normative samples; Oldham et al., 2018), therefore the focus on this task enabled the systematic integration of the findings. Future studies should use varied fMRI tasks to create a body of work examining how different facets of reward and loss processing are affected in people who use cannabis. Another limitation of the review is the exclusion of samples with comorbid mental health problems (e.g., schizophrenia, depression). While this approach enables the examination of cannabis-specific effects, the findings cannot be generalised to the most vulnerable people who use cannabis (Hasin and Walsh, 2020).

### 4.3 Conclusions

Overall, there exists largely non-significant evidence of brain alterations in cannabis users compared to controls, examined with the MID fMRI task. A subset of results reporting significant findings consistently identified significantly different *striatal* function during the anticipation of rewards and losses; and mixed results supporting associations between brain function and chronicity of cannabis use. Replication longitudinal neuroimaging studies of cannabis users are warranted to use robust metrics of substance use/mental health, and in relation to different types of rewards e.g., monetary, cannabis, and natural rewards (e.g., food). Such new evidence is required to identify with precision the neurobiology of reward processing in cannabis users and to enable comparison of the evidence in cannabis users with prominent neuroscientific theories of addiction.

## Author contributions

EB: Conceptualisation, Data curation, Formal analysis, Investigation, Methodology, Writing—original draught. GP: Writing—review & editing. SA: Writing—review & editing, Methodology. HT: Methodology, Writing—review & editing. VL: Writing—review & editing, Conceptualisation, Supervision.
