# Supplementary material for: Brain reward function in people who use cannabis: a systematic review
Source: Front Behav Neurosci. 2024 Feb 6;17:1323609. doi: 10.3389/fnbeh.2023.1323609 (PMC10877725; doi:10.3389/fnbeh.2023.1323609)
Supplement: Supplementary file 1 [file Data_Sheet_1.docx]

Supplementary Material

# Search Strategy

**Supplementary Table 1:** Search Strategy for PsycInfo, search on 13/06/2022

| **#** | **Search Terms** | **Results** |
| --- | --- | --- |
| **Concept 1: Cannabis** | | |
| **#1** | MM "Cannabis" OR MM "Cannabinoids" OR MM "Hashish" OR MM "Marijuana" OR MM "Cannabis Use Disorder" OR MM "Cannabidiol" | **12,644** |
| **#2** | Cannabis* OR CUD OR THC OR CBD OR Marijuana* OR hash* OR cannabinoid*; ti,ab | **28,796** |
| **#3** | #1 OR #2 | **29,670** |
| **Concept 2: Medicinal** | | |
| **#4** | DE “Functional Magnetic Resonance Imaging” OR DE “Neuroimaging” OR DE “Brain Connectivity” | **50,921** |
| **#5** | fMRI OR functional MRI OR functional magnetic resonance imag* OR functional neuroimag* OR BOLD OR blood oxygen level-dependent OR brain function* OR brain connect* OR neural connect* OR functional connect* OR brain activity OR neural activity OR functional activity; ti,ab | **233,945** |
| **#6** | #4 OR #5 | **244,970** |
| **Concept 3: Reward Processing** | | |
| **#7** | MM "Monetary Incentives" OR MM "Incentives" OR MM "Monetary Rewards" OR MM "Rewards" | **17,370** |
| **#8** | Monetary* OR incentiv* OR delay* OR MID OR MIDT OR reward* OR anticipat* OR receipt*; ti, ab | **202,247** |
| **#9** | #7 OR #8 | **203,412** |
| **#10** | #3 **AND** #6 **AND** #9 | **326** |

**Supplementary Table 2:** Search Strategy for MEDLINE, search on 26/06/2022

| **#** | **Search Terms** | **Results** |
| --- | --- | --- |
| **Concept 1: Cannabis** | | |
| **#1** | MH "Cannabis" OR MM "Marijuana Abuse" OR MM "Marijuana Smoking" | **31,809** |
| **#2** | Cannabis* OR CUD OR THC OR CBD OR Marijuana* OR hash* OR cannabinoid*; ti,ab | **72,505** |
| **#3** | #1 OR #2 | **76,247** |
| **Concept 2: Functional Neuroimaging** | | |
| **#4** | MM "Functional Neuroimaging+" OR MM "Neuroimaging" | **45,029** |
| **#5** | fMRI OR functional MRI OR functional magnetic resonance imag* OR functional neuroimag* OR BOLD OR blood oxygen level-dependent OR brain function* OR brain connect* OR neural connect* OR functional connect* OR brain activity OR neural activity OR functional activity; ti,ab | **811,643** |
| **#6** | #4 OR #5 | **829,769** |
| **Concept 3: Monetary Incentive Delayed Task** | | |
| **#7** | MH "Rewards" | **24,322** |
| **#8** | Monetary* OR incentiv* OR delay* OR MID OR MIDT OR reward* OR anticipat* OR receipt*; ti, ab | **869,777** |
| **#9** | #7 OR #8 | **874,209** |
| **#10** | #3 **AND** #6 **AND** #9 | **525** |

**Supplementary Table 3:** Search Strategy for CINAHL, search on 27/06/2022

| ***CINAHL*** *27/06/2022* | |  |
| --- | --- | --- |
| **#** | **Search Terms** | **Results** |
| **Concept 1: Cannabis** | | |
| **#1** | MH "Cannabis+" | **11,645** |
| **#2** | Cannabis* OR CUD OR THC OR CBD OR Marijuana* OR hash* OR cannabinoid*; ti,ab | **24,351** |
| **#3** | #1 OR #2 | **26,988** |
| **Concept 2: Cannabis** | | |
| **#4** | MM "Magnetic Resonance Imaging+" | **52,953** |
| **#5** | fMRI OR functional MRI OR functional magnetic resonance imag* OR functional neuroimag* OR BOLD OR blood oxygen level-dependent OR brain function* OR brain connect* OR neural connect* OR functional connect* OR brain activity OR neural activity OR functional activity; ti,ab | **94,460** |
| **#6** | #4 OR #5 | **141,611** |
| **Concept 3: Monetary Incentive Delayed Task** | | |
| **#7** | MH "Rewards" | **5,073** |
| **#8** | Monetary* OR incentiv* OR delay* OR MID OR MIDT OR reward* OR anticipat* OR receipt*; ti, ab | **194,993** |
| **#9** | #7 OR #8 | **195,331** |
| **#10** | #3 **AND** #6 **AND** #9 | **81** |

**Supplementary Table 4:** Search Strategy for PubMED, search on 27/06/2022

| ***PubMed*** *27/06/2022* | |  |
| --- | --- | --- |
| **#** | **Search Terms** | **Results** |
| **Concept 1: Cannabis** | | |
| **#1** | Cannabis* OR CUD OR THC OR CBD OR Marijuana* OR hash* OR cannabinoid*; ti,ab | **81,290** |
| **Concept 2: Functional Neuroimaging** | | |
| **#2** | fMRI OR functional MRI OR functional magnetic resonance imag* OR functional neuroimag* OR BOLD OR blood oxygen level-dependent OR brain function* OR brain connect* OR neural connect* OR functional connect* OR brain activity OR neural activity OR functional activity; ti,ab | **158,893** |
| **Concept 3: Monetary Incentive Delayed Task** | | |
| **#3** | Monetary* OR incentiv* OR delay* OR MID OR MIDT OR reward* OR anticipat* OR receipt* | **980,786** |
| **#4** | #3 **AND** #6 **AND** #9 | **208** |

**Supplementary Table 5:** Search Strategy for Scopus, search on 27/06/2022

| **#** | **Search Terms** | **Results** |
| --- | --- | --- |
| **Concept 1: Cannabis** | |  |
| **#1** | TITLE-ABS-KEY(Cannabis* OR CUD OR Marijuana* OR hash*) | **178,549** |
| **Concept 2: Functional Neuroimaging** | |  |
| **#2** | TITLE-ABS-KEY("fMRI" OR "functional magnetic resonance imaging" OR "functional MRI" OR (("brain imaging" OR neuroimag* OR BOLD OR "blood oxygen level-dependent" OR connectivity OR activity) AND functional)) | **623,133** |
| **Concept 3: Mindfulness** | |  |
| **#3** | TITLE-ABS-KEY(monetary* OR incentiv* OR delay* OR mid OR midt OR reward* OR anticipat* OR receipt*) | **2,430,121** |
| **#4** | #1 AND #2 AND #3 | **371** |

**Supplementary Table 6:** Search Strategy for Web of Science, search on 27/06/2022

| **#** | **Search Terms** | **Results** |
| --- | --- | --- |
| **Concept 1: Cannabis** | | |
| **#1** | TS=(Cannabis* OR CUD OR Marijuana* OR hash*) | **92,548** |
| **Concept 2: Functional Neuroimaging** | | |
| **#2** | TS=(fMRI OR functional MRI OR functional magnetic resonance imag* OR functional neuroimag* OR BOLD OR blood oxygen level dependent OR brain function* OR brain connect* OR neural connect* OR functional connect* OR brain activity OR neural activity OR functional activity) | [**1,206,641**](https://www-webofscience-com.ezproxy1.acu.edu.au/wos/woscc/summary/c8b5d84a-07f1-4de7-bb8c-56a7777ee91f-40583d62/relevance/1) |
| **Concept 3: Mindfulness** | | |
| **#3** | TS=(monetary* OR incentiv* OR delay* OR mid OR midt OR reward* OR anticipat* OR receipt*) | **1,849, 415** |
| **#4** | #1 AND #2 AND #3 | **324** |

# Overview of Monetary Rewards Used in the Literature

Supplementary Table 7-8 overviews the MID fMRI task parameters and trial times. The *monetary rewards* offered to participants to robustly engage in reward processing varied between studies. In all studies but one, monetary reward was offered *based on* successful trials when participants pressed the target trial button on time. The currencies used in the studies varied from euros (Enzi et al., 2015; Jager et al., 2013; van Hell et al., 2010) to US dollars (Filbey et al., 2013; Karoly et al., 2015; Yip et al., 2014) to pounds (Skumlien et al., 2022). *For consistency, US currency was reported to allow a standardised synthesis.* Rewards/deductions ranged from 10c to $5.00 per trial (Enzi et al., 2015; Jager et al., 2013; Karoly et al., 2015; Skumlien et al., 2022; van Hell et al., 2010; Yip et al., 2014). In 3 studies, a *total amount* of $40.00; van Hell et al., 2010), $51.00 (Nestor et al., 2010) or $19.00; Jager et al., 2013) was offered regardless of task performance. Additionally, 1 study did not report monetary rewards or reimbursements for participation (Nestor et al., 2020).

The *success rate* achievable by participants to maximise brain response to rewards and lack of rewards was set at 50% (n = 5), followed by 66% (n = 3), or it was not reported (Yip et al., 2014). The *order of the trials* varied between studies. In 4 studies, each trial was presented in pseudorandomised order (Enzi et al., 2015; Filbey et al., 2013; Nestor et al., 2010; Nestor et al., 2020), while 5 studies did not report any randomised trial order).

The literature measures MID fMRI *task performance* via accuracy (e.g., amount of money won from each trial), and percent correct (e.g., portion of money won from each trial).

# Overview of MID fMRI Tasks Duration

Only 2 studies reported *task duration*, which ranged from 7 minutes (Filbey et al., 2013) to 12 minutes (Yip et al., 2014) and it was not reported in the others. In all studies but 1 (Yip et al., 2014), the duration of the task was manipulated by adjusting the success rate based on the participant’s response time during practice trials (e.g., if the participants’ response time was too fast or too slow, the target duration was adjusted accordingly to increase or decrease the difficulty level of the task).

## Practice Runs

Practice runs completed in the scanner were reported by all but 2 studies to ensure that participants were trained for performing the task correctly (Nestor et al., 2020; Yip et al., 2014). The duration of the practice runs ranged from 2 to 7 minutes and was reported only by 2 studies (Filbey et al., 2013; Karoly et al., 2015). In 2 studies, practice runs were reported via the amount of trials complete (10 trials; Jager et al., 2013; van Hell et al., 2010)

## Task Trials for Each Contrast

The *total number of trials* varied widely: 27 (Nestor et al., 2010; Nestor et al., 2020), 55 (Yip et al., 2014), 66 (Skumlien et al., 2022), 72 (Jager et al., 2013; van Hell et al., 2010), and 144 (Enzi et al., 2015; Filbey et al., 2013; Karoly et al., 2015). The number of reward anticipation trials varied also: 9 (Nestor et al., 2010; Nestor et al., 2020), 22 (Yip et al., 2014), 28 (Skumlien et al., 2022), to 54 (Enzi et al., 2015; Filbey et al., 2013; Karoly et al., 2015). Reward anticipation trials and loss anticipation trials were inconsistent: from 9 (Nestor et al., 2010; Nestor et al., 2020) to 22 (Yip et al., 2014), and 54 (Enzi et al., 2015; Filbey et al., 2013; Karoly et al., 2015). Neutral anticipation and outcome trials ranged from 9 (Nestor et al., 2010; Nestor et al., 2020), to 11 (Yip et al., 2014), 36 (Enzi et al., 2015; Filbey et al., 2013; Karoly et al., 2015), and 38 (Skumlien et al., 2022). Two studies reported 72 trials without a breakdown by trial type (Jager et al., 2013; van Hell et al., 2010).

**Supplementary Table 7:** *Overview of Monetary Incentive Delay fMRI Characteristics*

| **Author (yr)** | **No. Trials** | **Randomisation** | **Possible Wins** | **Practice Runs** | **Hit Rate** |
| --- | --- | --- | --- | --- | --- |
| Enzi et al. (2015) | 144 | Yes | 10c, 60c or €3 | Yes | 66% |
| Filbey et al. (2013) | 144 | Yes | 20c, $1 or $5 | Yes | 66% |
| Jager et al. (2013) | 72 | - | €17 | Yes | 50% |
| Karoly et al. (2015) | 144 | - | 20c, $1 or $5 | Yes | 66% |
| Nestor et al. (2010) | 27 | Yes | 50c, -50c, or $0 | Yes | 50% |
| Nestor et al. (2020) | 27 | Yes | 50c, -50c, or $0 | - | 50% |
| Skumlien et al. (2022) | 66 | - | 50c or $0 | Yes | 50% |
| van Hell et al. (2010) | 72 | - | €36 | Yes | 50% |
| Yip et al. (2014) | 55 | - | 50c, $1, or $5 | - |  |

| **Author (yr)** | **Cue** | **Anticipation** | **Target** | **Feedback** | **Delay** | **End Fixation** |
| --- | --- | --- | --- | --- | --- | --- |
| Enzi et al. (2015) | 250ms | 3740 – 4240 ms | 160 – 360ms | 1650ms | 1500 – 2200ms |  |
| Filbey et al. (2013) | 250ms | - | 166 – 435ms | 1650ms | 1165 – 1934ms | - |
| Jager et al. (2013) | 3000 – 10000 ms | - | 3000 – 10000 ms | - | - | - |
| Karoly et al. (2015) | 250ms | - | 166 – 435ms | 1650ms | 1165 – 1934ms |  |
| Nestor et al. (2010) | 2000 – 8000ms | - | 400ms | 1500ms | - | 2000 – 8000ms |
| Nestor et al. (2020) | 2000 – 8000ms | - | 400ms | 1500ms | - | 2000 – 8000ms |
| Skumlien et al. (2022) | 500ms | 2000 – 4000ms | - | - | - | - |
| van Hell et al. (2010) | - | - | - | - | - | - |
| Yip et al. (2014) | 1000ms | - | - | 1200ms | 3000 – 5000ms | - |
| Note. ms = milliseconds | | | | | | |

**Supplementary Table 8:** *Overview of Monetary Incentive Delay Trial Times*

# Overview of Contrasts used to Examine Brain Function Durin the MID fMRI Task

Supplementary Table 9 overviews the MID fMRI task contrasts used to examine brain function in the literature to date. Brain function was examined most commonly using the *contrast reward anticipation > neutral anticipation* (n = 7), followed by *loss anticipation > neutral anticipation* (n = 6), and *reward feedback > neutral feedback* (n = 4). Other contrasts were reported by 3 studies or less. They included: *reward anticipation > loss anticipation* (n = 2); *reward feedback > loss feedback* (n = 2); *reward feedback > neutral feedback* (n = 1). Other studies reported stand-alone condition for *reward anticipation* (n = 3), *neutral anticipation* (n=3), *loss anticipation* (n = 2), *reward feedback* (n=2), *loss feedback* (n = 1), *avoidance loss feedback* (n=1), and *neutral feedback* (n = 2).

**Supplementary Table 9:** Overview of Monetary Incentive Delay fMRI Task contrasts examined in the literature to date.

| **Author (yr)** | **Anticipation of** |  |  | **Anticipation of conditions** | | | |
| --- | --- | --- | --- | --- | --- | --- | --- |
|  | **Reward vs. Neutral** | **Loss vs. Neutral** | **Reward vs. Loss** | **Reward** | | **Loss** | **Neutral** |
| Skumlien et al. (2022) | reward>neutral | - | - | - | | - | - |
| Nestor et al. (2020) | - | loss>neutral | **-** | ✓ | | ✓ | ✓ |
| Enzi et al. (2015) | reward>neutral | loss>neutral | reward>loss | - | | - | - |
| Karoly et al. (2015) | reward>neutral | loss>neutral | - | - | | - | - |
| Yip et al. (2014) | reward>neutral | loss>neutral | - | - | | - | - |
| Filbey et al. (2013) | reward>neutral | loss>neutral | reward>loss loss> reward | - | | - | - |
| Jager et al. (2013) | reward>neutral | - | - | ✓ | | - | ✓ |
| Nestor et al. (2010) | - | loss>neutral | - | ✓ | | ✓ | ✓ |
| van Hell et al. (2010) | reward>neutral | - | - | - | | - | - |
|  | **Feedback of** | | | | **Feedback of conditions** | | |
|  | **Reward vs. Neutral** | **Loss vs. Neutral** | **Reward vs. Loss** | **Reward** | | **Loss** | **Neutral** |
| Skumlien et al. (2022) | reward>neutral | - | ^-^ | - | | ^-^ | - |
| Nestor et al. (2020) | - | - | - | - | | - | - |
| Enzi et al. (2015) | reward>neutral | loss>neutral | reward>loss | ✓ | | ✓ | ✓ |
| Karoly et al. (2015) | - | - | - | - | | - | - |
| Yip et al. (2014) | reward>neutral | loss>neutral | - | - | | - | - |
| Filbey et al. (2013) | - | - | - | ✓ | | ✓ | - |
| Jager et al. (2013) | - | - | - | - | | - | - |
| Nestor et al. (2010) | - | - | - | - | | ✓(avoidance) | ✓ |
| van Hell et al. (2010) | reward>neutral | - | - | - | | - | - |

# Overview of Recruitment Source and Study Locations

The recruitment source to find participants was described in all but two studies (Enzi et al., 2015; Jager et al., 2013). In 4 studies, samples were recruited from the general community (Filbey et al., 2013; Nestor et al., 2010; Skumlien et al., 2022; van Hell et al., 2010). In individual studies, cannabis participants were recruited from an outpatient treatment program (Yip et al., 2014), a drug treatment program (Nestor et al., 2020), and a youth program (Karoly et al., 2015). The location of the studies was described in all but 3 studies (Karoly et al., 2015; van Hell et al., 2010; Yip et al., 2014) and included Germany (Enzi et al., 2015), New-Mexico (Filbey et al., 2013), Ireland (Nestor et al., 2010; Nestor et al., 2020), Netherlands/USA (Jager et al., 2013), and England (Skumlien et al., 2022). The 3 studies that did not report locations, it was extrapolated from the location of the lead author that the studies were based in, which included the USA (Karoly et al., 2015; Yip et al., 2014) and the Netherlands (van Hell et al., 2010).

# Metrics of Cannabis Use

Cannabis exposure was measured inconsistently in the literature. In 4 of the 9 studies, the duration of cannabis use was reported for an average of 9 years (range; 6-14 years). In 5 of the 9 studies participants used *cannabis* on an average of 27 days per month (range; 16-28 days). The duration of abstinence from cannabis prior to the fMRI scan was reported in 6 studies and ranged from 3 hours to 35 days. Cannabis dosage was measured in an inconsistent fashion, including joints/week, gram/week, and hits/day.

All but 2 studies performed urine toxicology analysis to confirm cannabis use and/or abstinence (Enzi et al., 2015; Filbey et al., 2013; Jager et al., 2013; Nestor et al., 2010; Nestor et al., 2020; van Hell et al., 2010; Yip et al., 2014). Of these, 1 study also utilised urine and blood toxicology to measure exposure to any other psychoactive substance (Enzi et al., 2015). This study also reported 11-nor-9-carboxy-Δ9-tetrahydrocannabinol (THC-COOH) levels.

# Assessment of the Methodological Quality of the Reviewed Literature

Supplementary Table 10 outlines the risk of bias of the included studies, which ranged from low, moderate, to high quality. Less than half of the studies (n = 4) were rated as high quality, with an average quality rating of 6 out of 7 (Filbey et al., 2013; Nestor et al., 2010; Nestor et al., 2020; Skumlien et al., 2022). More than half the studies (n = 5) were rated to be of moderate quality, with an average score of 4.5 of 7. The most consistently reported issues were: the lack of reporting of inclusion criteria (Enzi et al., 2015; Karoly et al., 2015; van Hell et al., 2010; Yip et al., 2014), the study setting (e.g., where the study took place; Enzi et al., 2015; Jager et al., 2013; Karoly et al., 2015; van Hell et al., 2010), measurement of criteria assessing cannabis use (Enzi et al., 2015; Jager et al., 2013; Karoly et al., 2015; van Hell et al., 2010; Yip et al., 2014), and accounting for potential confounders in data analysis (Jager et al., 2013; Nestor et al., 2020; Yip et al., 2014).

**Supplementary Table 10:** Overview of Risk of Bias of the MID fMRI Task Literature in Cannabis Users, as per the Joanna Briggs Institute Critical Appraisal Checklist for Cross-sectional Studies

| **Author (yr)** | **Study quality** | **(I)**  **Inclusion criteria clearly defined?** | **(II)**  **Subjects/setting described in detail?** | **(III)**  **Objective, standard criteria to measure condition?** | **(IV)**  **Confounders identified?** | **(V)**  **Strategies to deal with confounders stated?** | **(VI)**  **Outcomes measure valid/reliable?** | **(VII)**  **Appropriate statistical analysis?** | **Mean rating across criteria 0-to-1** |
| --- | --- | --- | --- | --- | --- | --- | --- | --- | --- |
| Enzi et al. (2015) | Moderate | 0 | 0 | 0 | 1 | 1 | 1 | 1 | 0.6 |
| Filbey et al. (2013) | High | 1 | 1 | 1 | 1 | 1 | 1 | 1 | 1 |
| Jager et al. (2013) | Moderate | 1 | 0 | 0 | 1 | 0 | 1 | 1 | 0.6 |
| Karoly et al. (2015) | Moderate | 0 | 0.5 | 0 | 1 | 1 | 1 | 1 | 0.6 |
| Nestor et al. (2010) | High | 1 | 1 | 1 | 1 | 1 | 1 | 1 | 1 |
| Nestor et al. (2020) | High | 1 | 1 | 1 | 1 | 0 | 1 | 1 | 0.9 |
| Skumlien et al. (2022) | High | 1 | 1 | 1 | 1 | 1 | 1 | 1 | 1 |
| van Hell et al. (2010) | Moderate | 0 | 0.5 | 0 | 1 | 1 | 1 | 1 | 0.6 |
| Yip et al. (2014) | Moderate | 0.5 | 1 | 0.5 | 1 | 0 | 1 | 1 | 0.7 |

**References**

Enzi, B., Lissek, S., Edel, M. A., Tegenthoff, M., Nicolas, V., Scherbaum, N., Juckel, G., & Roser, P. (2015). Alterations of monetary reward and punishment processing in chronic cannabis users: an FMRI study. *PLoS One*, *10*(3), e0119150. <https://doi.org/10.1371/journal.pone.0119150>

Filbey, F. M., Dunlop, J., & Myers, U. S. (2013). Neural effects of positive and negative incentives during marijuana withdrawal. *PLoS One*, *8*(5), e61470. <https://doi.org/10.1371/journal.pone.0061470>

Jager, G., Block, R. I., Luijten, M., & Ramsey, N. F. (2013). Tentative evidence for striatal hyperactivity in adolescent cannabis-using boys: A cross-sectional multicenter fMRI study. *Journal of Psychoactive Drugs*, *45*(2), 156-167. <https://doi.org/10.1080/02791072.2013.785837>

Karoly, H. C., Bryan, A. D., Weiland, B. J., Mayer, A., Dodd, A., & Ewing, S. W. F. (2015). Does incentive-elicited nucleus accumbens activation differ by substance of abuse? An examination with adolescents. *Developmental Cognitive Neuroscience*, *16*, 5-15. <https://www.ncbi.nlm.nih.gov/pmc/articles/PMC4657439/pdf/main.pdf>

Nestor, L., Hester, R., & Garavan, H. (2010). Increased ventral striatal BOLD activity during non-drug reward anticipation in cannabis users. *Neuroimage*, *49*(1), 1133-1143. <https://doi.org/10.1016/j.neuroimage.2009.07.022>

Nestor, L. J., Behan, B., Suckling, J., & Garavan, H. (2020). Cannabis-dependent adolescents show differences in global reward-associated network topology: A functional connectomics approach. *Addiction biology*, *25*(2), e12752. <https://doi.org/10.1111/adb.12752>

Skumlien, M., Mokrysz, C., Freeman, T. P., Wall, M. B., Bloomfield, M., Lees, R., Borissova, A., Petrilli, K., Carson, J., Coughlan, T., Ofori, S., Langley, C., Sahakian, B. J., Curran, H. V., & Lawn, W. (2022). Neural responses to reward anticipation and feedback in adult and adolescent cannabis users and controls. *Neuropsychopharmacology*, *47*(11), 1976-1983. <https://doi.org/10.1038/s41386-022-01316-2>

van Hell, H. H., Vink, M., Ossewaarde, L., Jager, G., Kahn, R. S., & Ramsey, N. F. (2010). Chronic effects of cannabis use on the human reward system: An fMRI study. *European Neuropsychopharmacology*, *20*(3), 153-163. <https://doi.org/10.1016/j.euroneuro.2009.11.010>

Yip, S. W., DeVito, E. E., Kober, H., Worhunsky, P. D., Carroll, K. M., & Potenza, M. N. (2014). Pretreatment measures of brain structure and reward-processing brain function in cannabis dependence: An exploratory study of relationships with abstinence during behavioral treatment. *Drug Alcohol Dependence*, *140*, 33-41. <https://doi.org/10.1016/j.drugalcdep.2014.03.031>
